# Vaping-Associated Lung Injury With Suspected Diffuse Alveolar Haemorrhage: A Case Report

**DOI:** 10.7759/cureus.82799

**Published:** 2025-04-22

**Authors:** Odunayo Yusuf, Christian Greenstreet

**Affiliations:** 1 Infectious Diseases, Manchester University NHS Foundation Trust, Manchester, GBR; 2 Acute Internal Medicine, Manchester University NHS Foundation Trust, Manchester, GBR

**Keywords:** alveolar, anaemia, e-cigarette, evali, haemorrhage, lung, pulmonary, vaping

## Abstract

E-cigarette or vaping product use-associated lung injury (EVALI) is a recognised condition with diverse presentations, including acute lung injury and, rarely, diffuse alveolar haemorrhage (DAH). This case report describes a rare instance of suspected DAH associated with herbal-based vape product use, highlighting diagnostic challenges and effective management.

A 44-year-old patient with Hashimoto’s thyroiditis, asthma and six-week use of herbal or plant-based vaping solutions presented with one week of cough, fever and haemoptysis. Examination revealed respiratory distress, bilateral crackles and wheezing. Arterial blood gas showed type 1 respiratory failure (PaO₂ 10.2 kPa, FiO₂ 0.7), and chest imaging displayed bilateral interstitial opacities suggestive of DAH. Differentials, including pneumonia, pulmonary embolism, vasculitis, Goodpasture’s syndrome and malignancy, were evaluated. Bronchoscopy confirmed left upper lobe haemorrhage, with bronchoalveolar lavage showing progressive red blood cells, supporting suspected DAH. Extensive autoimmune, infectious and neoplastic workup (e.g., antinuclear antibody, viral polymerase chain reaction (PCR), cultures) was negative. Empirical antibiotics and antifungals were stopped after negative cultures. On day 3, suspecting DAH, high-dose intravenous methylprednisolone (1,000 mg/day for three days) was initiated for immune-mediated inflammation, yielding rapid clinical and radiological improvement. EVALI with suspected DAH was diagnosed by exclusion post-workup, and vaping cessation was advised. At three months, the patient remained stable without recurrence.

This case underscores EVALI as a key differential in vaping patients with haemoptysis and bilateral infiltrates when other aetiologies are excluded. A thorough vaping history, comprehensive workup and prompt corticosteroid therapy can yield favourable outcomes. The rare link between DAH and EVALI warrants further research into its prevalence and management.

## Introduction

Diffuse alveolar haemorrhage (DAH) is a rare and potentially life-threatening condition characterized by dyspnoea, haemoptysis, diffuse radiographic abnormalities and anaemia, arising from diverse aetiologies such as primary lung diseases, infections, renal or cardiac disorders and systemic autoimmune diseases [1]. Immune-mediated DAH, accounting for approximately 35% of cases, is predominantly associated with ANCA-associated vasculitides, including microscopic polyangiitis and granulomatosis with polyangiitis [2].

Infections, notably *Staphylococcus aureus* (including methicillin-resistant and Panton-Valentine leukocidin-producing strains), are less common causes but may present with haemorrhagic pneumonia, particularly in patients with acute infectious symptoms [2]. DAH carries a significant in-hospital mortality rate of 24.7% [3].

This case report describes a rare presentation of suspected DAH within the context of e-cigarette or vaping product use-associated lung injury (EVALI), a condition often manifesting as acute lung injury (ALI) with features like hypoxemia and bilateral infiltrates [4]. Since its recognition in 2019, EVALI has been increasingly diagnosed, with early case series reporting diverse presentations, including spontaneous pneumothorax, eosinophilic pneumonia, hypersensitivity pneumonitis and, rarely, pulmonary haemorrhage [4,5]. Notable cases of suspected DAH linked to vaping include severe haemoptysis requiring intensive care, often associated with herbal or plant-based vape products, though confirmed cases remain sparse [6,7]. The overlap between suspected DAH and ALI in EVALI underscores the disease’s heterogeneous pathology, necessitating careful diagnostic evaluation to distinguish specific patterns.

E-cigarettes, popularised since the 2000s, particularly among younger populations in Western countries, deliver aerosols containing nicotine, THC, synthetic compounds and herbal derivatives [4,5]. Substances such as vitamin E acetate, identified in many EVALI cases, have been shown to disrupt alveolar surfactant, induce oxidative stress and trigger proinflammatory responses, leading to epithelial and endothelial injury, capillary leakage and, in rare instances, alveolar haemorrhage [5,8]. These toxic effects contribute to EVALI’s varied manifestations, including the suspected DAH observed in this case [8].

## Case presentation

A 44-year-old patient with a documented medical history of Hashimoto’s thyroiditis and asthma presented to the emergency department with a one-week history of cough, fever and haemoptysis. The patient, a former smoker, was an active user of e-cigarettes and had transitioned, six weeks prior to presentation, to herbal or plant-based vaping solutions purchased from an online vendor. Upon clinical examination, they exhibited signs of respiratory distress, with auscultation revealing bilateral crackles and wheezing.

Arterial blood gas analysis indicated type 1 respiratory failure, with a partial pressure of oxygen (PaO₂) of 10.2 kPa on a fraction of inspired oxygen (FiO₂) of 0.7, suggesting severe hypoxemia. Laboratory findings demonstrated anaemia, mild neutrophilia and a raised C-reactive protein (CRP). However, eosinophil count, coagulation screen and biochemistry profile were unremarkable (Tables 1-3).

**Table 1:** Arterial blood gas analysis BE, base excess; FiO₂, fraction of inspired oxygen; pCO₂, partial pressure of carbon dioxide; pH, potential of hydrogen; pO₂, partial pressure of oxygen

Parameter	Results
FiO_2_	0.7
pH	7.42
pO_2_	10.2 kPa
pCO_2_	4.4 kPa
BE	-2
Lactate	0.6 mmol/L
Bicarbonate	22 mmol/L
Haemoglobin	69 g/L

**Table 2:** Haematology: blood count and coagulation screen APTT, activated partial thromboplastin time; CRP, C-reactive protein; ESR, erythrocyte sedimentation rate; INR, international normalised ratio; PT, prothrombin time; WBC, white blood cells

Parameters	Results
Haemoglobin	74 g/L
WBC	10.6 × 10^9^/L
Neutrophil	9.7 × 10^9^/L
Eosinophil	0.1 × 10^9^/L
Platelet count	329 × 10^9^/L
CRP	36 mg/L
ESR	18 mm/hour
PT	13.3 seconds
APTT	29.7 seconds
INR	1.3
Fibrinogen	2.9 g/L
Lupus anticoagulant	Negative

**Table 3:** Biochemistry profile

Parameters	Results
Sodium	141 mmol/L
Potassium	3.5 mmol/L
Chloride	107 mmol/L
Creatinine	35 µmol/L
Urea	2.7 mmol/L
Alanine aminotransferase	13 IU/L
Total bilirubin	33 µmol/L
Albumin	37 g/L
Alkaline phosphatase	53 IU/L
Calcium	2.21 mmol/L
Phosphate	1.10 mmol/L
Magnesium	0.75 mmol/L
Procalcitonin	0.05 ng/mL

**Figure 1:**
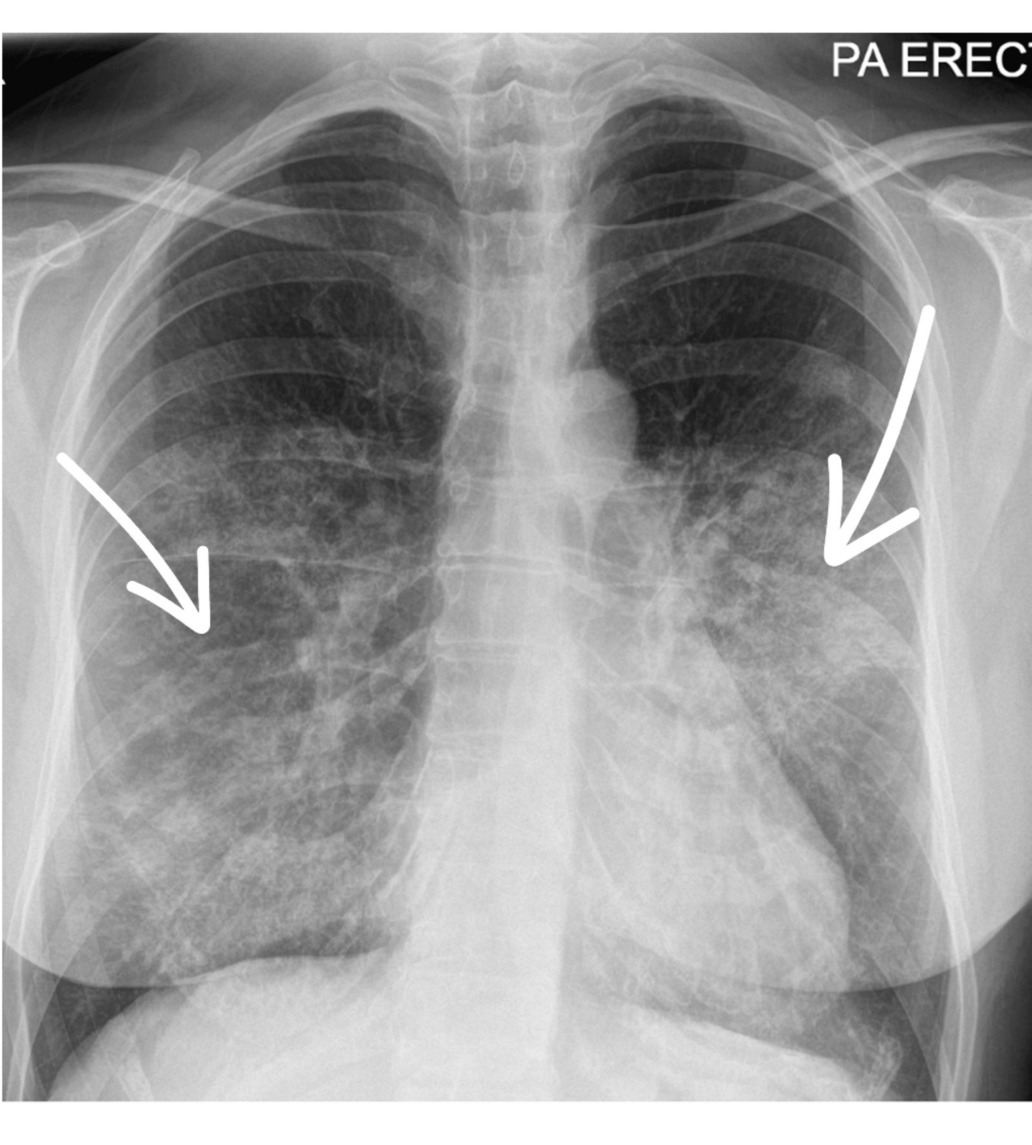
Chest X-ray on admission Posteroanterior chest X-ray with arrows indicating bilateral diffuse opacities, predominantly involving the lower lung zones.

**Figure 2:**
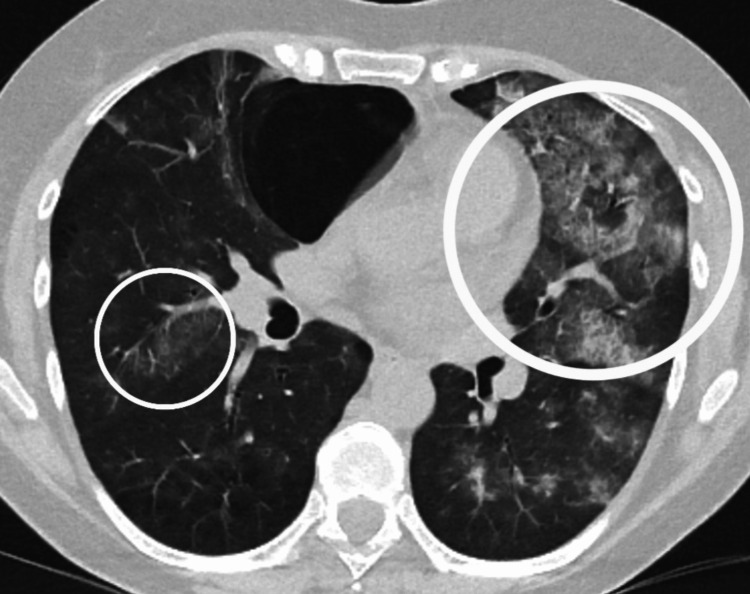
Computed tomography of the thorax on admission Axial CT thorax image showing encircled areas of bilateral interstitial ground-glass opacities.

A urine dipstick test was negative for blood and protein. Initial imaging via chest radiography (Figure 1), conducted upon admission, revealed diffuse interstitial shadowing. A subsequent CT thorax revealed extensive bilateral interstitial opacities, which the reporting radiologist noted as suggestive of possible DAH in the context of haemoptysis (Figure 2).

Given the unclear aetiology underlying the patient’s clinical presentation, a comprehensive diagnostic workup, encompassing infectious and immunological screens, was promptly initiated. Concurrently, the patient was started on an empirical treatment regimen consisting of broad-spectrum antibiotics (intravenous co-amoxiclav and clarithromycin) and an antifungal agent (voriconazole) pending infectious workup, as haemoptysis and infiltrates raised concern for infection. Potential toxicological aetiologies were also considered, prompting a detailed exploration of the patient’s vaping history at this juncture.

**Figure 3:**
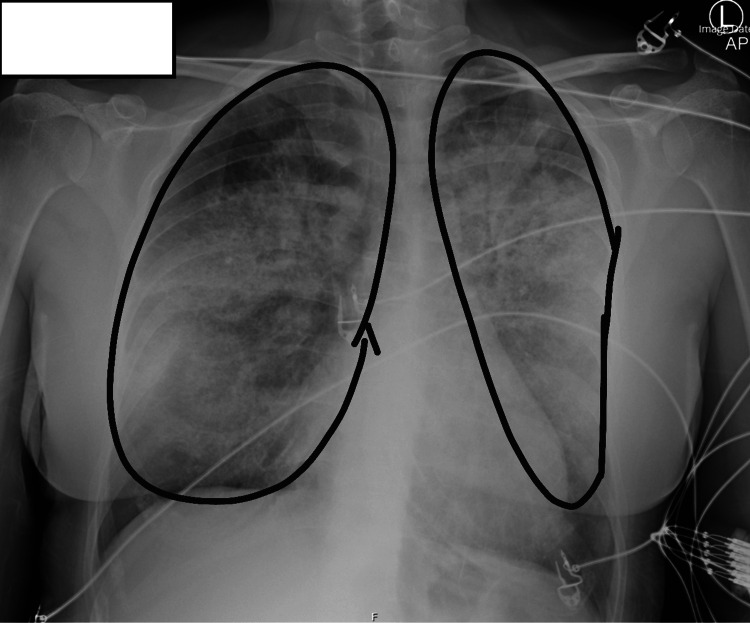
Day 2 chest X-ray Chest X-ray with encircled areas indicating worsening bilateral diffuse airway opacities, now involving the upper lobes compared to the admission radiograph (Figure 1).

**Figure 4:**
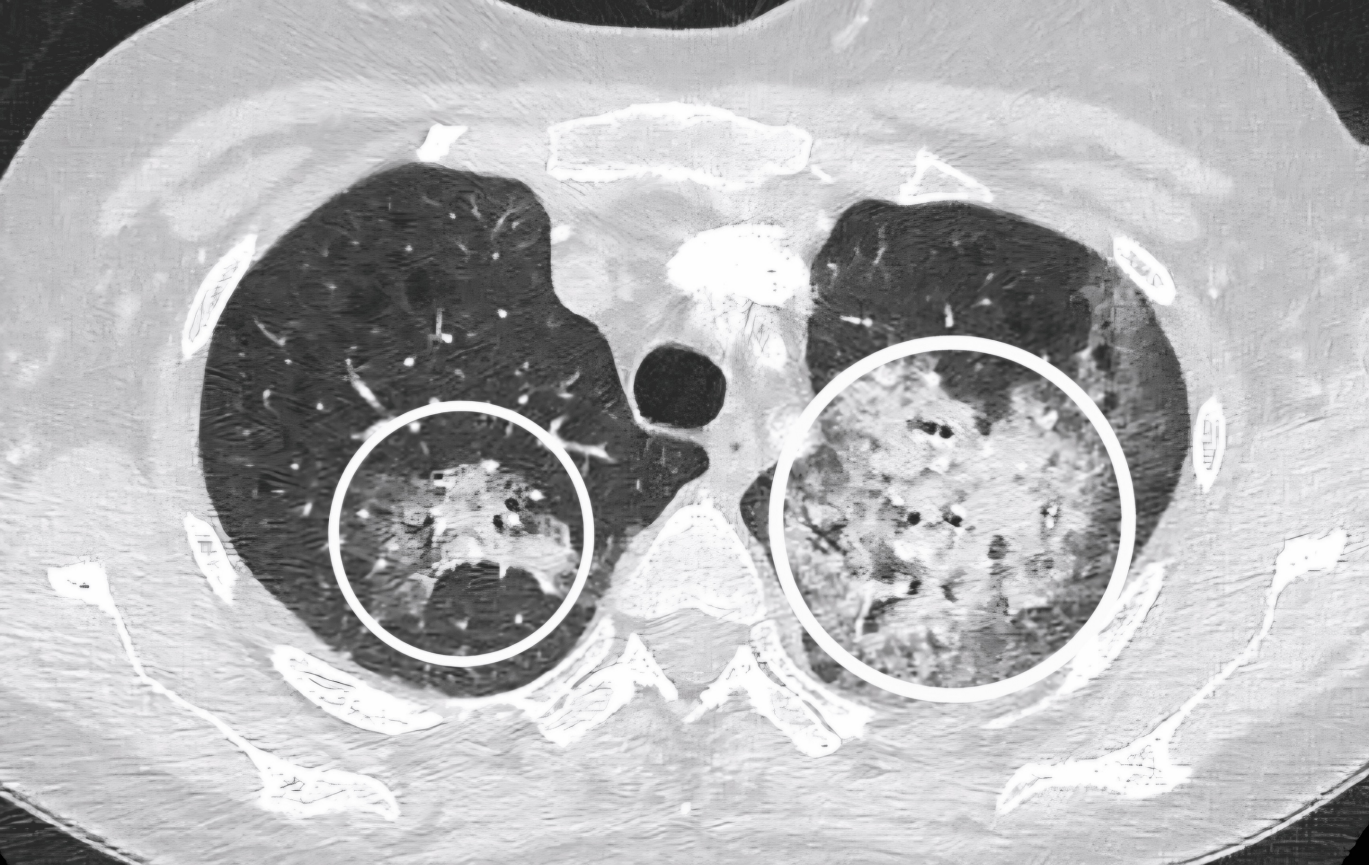
Computed axial tomography pulmonary angiogram Encircled areas highlight the progression of interstitial ground-glass opacities observed on the initial non-contrast CT thorax (Figure 2). No significant filling defects were identified in the bronchial or segmental pulmonary arteries to suggest pulmonary embolism.

By the second day of hospital admission, the patient’s hypoxia and respiratory distress had deteriorated further. A repeat chest X-ray revealed a progression of interstitial opacities (Figure 3). Additionally, a computed tomography pulmonary angiogram (CTPA) was conducted, effectively excluding the presence of a main stem or segmental pulmonary embolus (Figure 4). Consequently, the patient was transferred to the intensive care unit (ICU) for enhanced monitoring and ventilatory support, as well as to facilitate bronchoscopy.

Bedside bronchoscopy with bronchoalveolar lavage (BAL) was performed in the ICU, revealing an active haemorrhage in the left upper lobe. Analysis of three sequential BAL aliquots demonstrated a progressive increase in red blood cell content and scattered macrophages consistent with DAH suggestive of suspected DAH (as shown in the subsequent table); however, no images were recorded. Documentation regarding the evaluation of other bronchial segments was not available in the clinical records. 

An extensive autoimmune, oncologic and infectious disease workup, encompassing an extended respiratory viral panel, bacterial cultures, mycobacterial tuberculosis polymerase chain reaction (PCR) and atypical pneumonia screens, yielded negative results (Tables 4-6).

**Table 4:** Autoimmune diagnostic evaluation of diffuse alveolar haemorrhage ANA, anti-nuclear antibody; anti-GBM, anti-glomerular basement membrane antibody; anti-ds-DNA, double stranded-deoxyribonucleic acid; anti-MPO, anti-myeloperoxidase antibody; anti-PR3, anti-proteinase 3 antibody; RF, rheumatoid factor

Test	Result
ANA	Negative
Anti-ds-DNA	Negative
RF	<10 (0-13)
Anti-GBM IgG	<0.2 (<0.9)
Anti-PR3	<0.2 (<0.9)
Anti-MPO	<0.2 (<0.9)
PM Scl-75	Negative
PM Scl-100	Negative
Mi-2 alpha	Negative
Mi-2 beta	Negative
ENA Jo-1	Negative
Anti-Ro	Negative
Anti-La	Negative
Anti-Ku	Negative
Anti-CN1A	Negative

**Table 5:** Infectious diagnostic evaluation of diffuse alveolar haemorrhage AFB, acid-fast bacilli; Abs, antibodies; BAL, bronchoalveolar lavage; COVID, coronavirus disease; ELISA, enzyme-linked immunosorbent assay; HIV, human immunodeficiency virus; IgG, immunoglobulin G; MRSA, methicillin-resistant *Staphylococcus aureus*; PCP, *Pneumocystis* pneumonia; PCR, polymerase chain reaction; RSV, respiratory syncytial virus

Test	Result
Blood cultures	Negative fungal, bacterial, AFB
Sputum cultures	Negative fungal, bacterial, AFB
Urine streptococcal antigen	Negative
Urine legionella antigen	Negative
Mycoplasma PCR	Negative
MRSA	Negative
HIV 1 and 2 Abs	Non-reactive
HIV P24 antigen	Negative
COVID, influenza A/B and RSV	Negative
Extended respiratory viral PCR	Negative
PCP PCR (BAL)	Negative
*Aspergillus* antigen	Negative
*Aspergillus* galactomannan Ag ELISA	Negative
IgG *Aspergillus* Ag	20 (0-40)
*Mycobacterium tuberculosis* culture (sputum and BAL)	Negative
Procalcitonin	0.05 ng/mL

**Table 6:** Neoplastic diagnostic evaluation of diffuse alveolar haemorrhage BAL, bronchoalveolar lavage; RBCs, red blood cells

Investigation	Result
Peripheral blood smear	Negative for malignant cells
Flow cytometry	Negative for malignant cells
Cytology BAL	RBCs predominance and scattered macrophages; no malignant cells seen

On the third day of admission, given the bronchoscopy findings and the suspicion of DAH based on haemoptysis, bronchoscopic left upper lobe haemorrhage and BAL findings (Table 7), with lack of clinical improvement despite antimicrobial therapy and largely negative initial infectious workup suggesting a non-infectious cause, the patient was commenced on a three-day course of intravenous methylprednisolone at a dosage of 1,000 mg daily, followed by a tapering regimen of oral prednisolone targeting a presumed immune-mediated or inflammatory process. With an infectious aetiology largely excluded at this point, initiation of immunosuppressive therapy with corticosteroids was considered appropriate while awaiting the results of a comprehensive immunologic workup. 

**Table 7:** Bronchoscopy and bronchoalveolar lavage (BAL) findings

Parameter	Result
Bronchoscopy	Active haemorrhage in the left upper lobe; other segments not documented
BAL aliquot 1	Red blood cells present, scattered macrophages
BAL aliquot 2	Increased red blood cell content
BAL aliquot 3	Further increase in red blood cell content
Hemosiderin-laden macrophages	None reported
Cytology	No malignant cells

**Figure 5:**
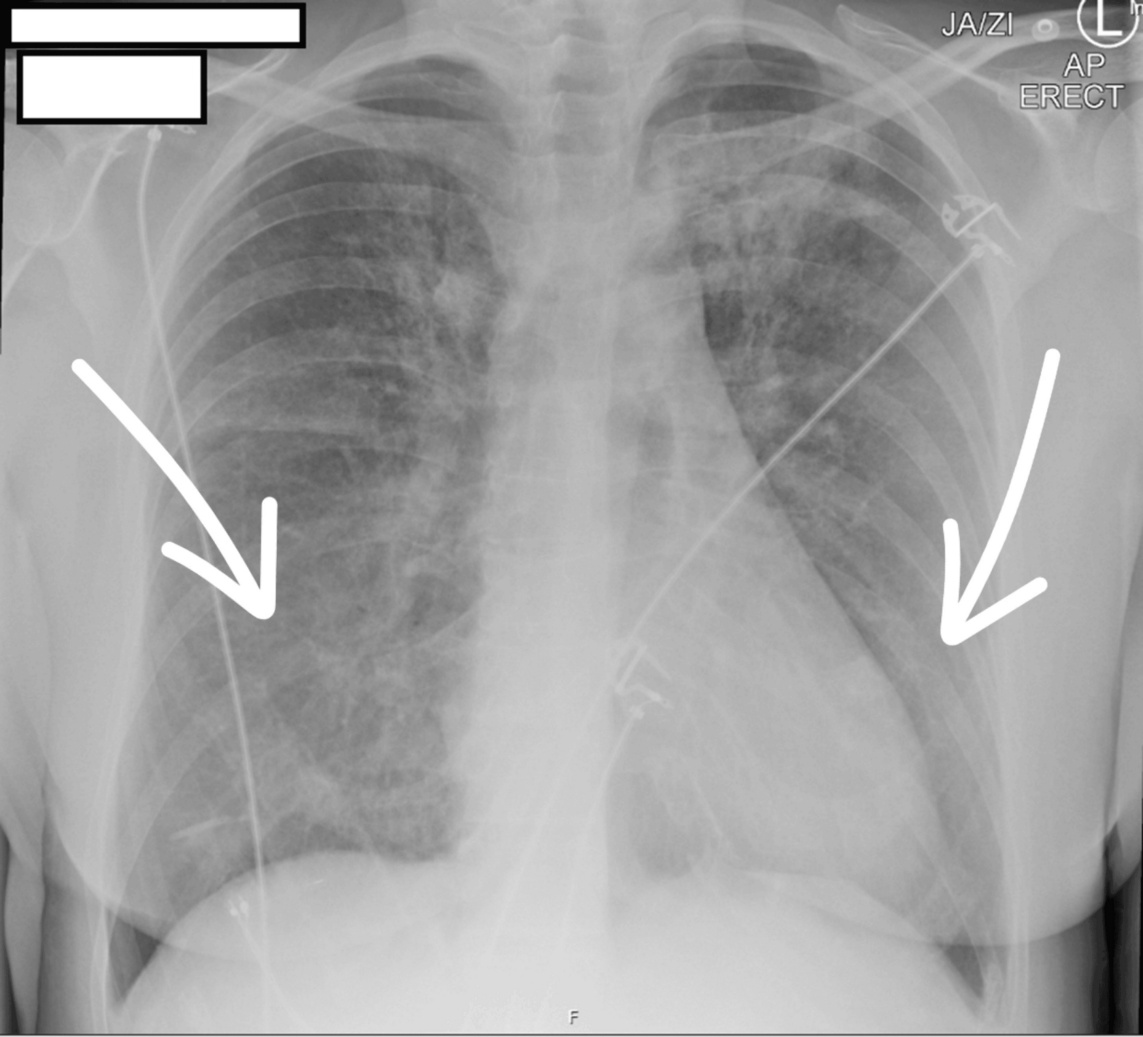
Day 5 chest X-ray This radiograph shows improving bilateral airway opacities, with arrows highlighting areas of notable change compared to imaging performed three days earlier (Figure 3).

Subsequently, the patient exhibited both clinical and radiological improvement (Figure 5). This rapid response to corticosteroids, alongside negative infectious results, supported an inflammatory process, prompting further evaluation for EVALI. In the absence of positive culture results, antibiotic and antifungal therapies were discontinued, and the patient’s care was transitioned to the medical high-dependency unit. 

The patient’s swift clinical and radiological improvement following empiric corticosteroids, combined with negative immunological and infectious investigations (Tables 4-5), confirmed an underlying inflammatory process. Following multidisciplinary consultations with specialists in respiratory medicine, rheumatology and microbiology, and given the absence of definitive immunological or infectious findings, the patient was diagnosed with EVALI, characterised by suspected DAH. The suspicion of DAH, initially raised on day 3 by haemoptysis, bronchoscopic identification of left upper lobe haemorrhage, and BAL showing a progressive increase in red blood cells (Table 7), was supported by the exclusion of other causes, while concurrent type 1 respiratory failure and bilateral interstitial opacities were consistent with the ALI pattern frequently observed in EVALI, enabling clinicians to recognise similar presentations [6].

The patient received comprehensive counselling on the diagnosis, with strong recommendations to permanently discontinue e-cigarette use, and was discharged with clear guidance to avoid vaping. At the three-month follow-up, the patient remained clinically stable, reporting no recurrence of symptoms.

## Discussion

The diagnosis of DAH typically represents a diagnostic challenge in acute type 1 respiratory failure, with over a hundred causes previously described. The diagnostic evaluation requires prompt identification of the underlying aetiology and institution of targeted therapy [2,5]. The most common aetiologies include immune-related conditions, cardiac failure and a wide range of infections. Consequently, the diagnostic workup is typically extensive and comprehensive [2].

This case underscores the diagnostic complexity of EVALI, particularly in distinguishing between ALI and DAH as manifestations of the disease. EVALI typically presents with ALI-like features, including acute hypoxemic respiratory failure, bilateral pulmonary infiltrates and non-cardiogenic pulmonary oedema, resembling the clinical criteria for ALI or acute respiratory distress syndrome (ARDS) [8,9]. However, DAH is characterised by alveolar bleeding, haemoptysis and progressive red blood cell accumulation in BAL and is a rarer but documented pattern within EVALI’s heterogeneous spectrum [6,10]. In the present case, the patient’s haemoptysis, bronchoscopic evidence of left upper lobe haemorrhage and BAL findings suggestive of alveolar bleeding prompted suspicion of DAH, yet the concurrent hypoxemia and bilateral infiltrates align with ALI’s broader phenotype. This overlap reflects EVALI’s variable pathology, where DAH may coexist with or mimic ALI, necessitating careful clinical correlation to guide diagnosis and management [6].

EVALI manifests as a diverse spectrum of pneumonitis patterns, including eosinophilic pneumonia, organising pneumonia, hypersensitivity pneumonitis, lipoid pneumonia and, less commonly, DAH [11]. Histologically, the most characteristic feature is the presence of lipid-laden pulmonary alveolar macrophages, often with vacuolisation, accompanied by vacuolated pneumocytes, findings indicative of chemical-induced pneumonitis [11].

These findings are non-specific and may arise from various causes, including drugs, irritative inhalants, gastric acid aspiration and viral infections. Consequently, obtaining a thorough history, considering the clinical context and excluding other potential etiologic conditions with similar presentations are crucial [11,12].

This case report highlights a rare presentation of suspected DAH associated with vaping product use, ultimately diagnosed as EVALI by exclusion. However, as with all case reports, the findings are inherently limited in scope and may not be generalisable to broader populations. The patient’s unique profile, including the use of plant-based vaping solutions, underlying asthma and rapid steroid response, represents a specific scenario that may not typify EVALI or vaping-related DAH. The rarity of DAH as a manifestation of EVALI, combined with the heterogeneity of vaping exposures, underscores the need for larger cohort studies or case series to establish its prevalence, risk factors and long-term outcomes. Such research is essential to clarify the spectrum of vaping-related lung injuries and inform standardised management strategies.

The high prevalence of THC, vitamin E acetate and various flavouring agents has yet to be fully substantiated, and further research is needed to identify the common toxic element [12]. Most commercially available e-cigarette solvents are mixed with a base of vegetable glycerine, a highly lipid-soluble substance. This increases the likelihood of inflammatory substances and solvents reaching the lung parenchyma, where they can induce damage. These substances elicit inflammation of the pulmonary epithelium and endothelium, leading to collagen deposition, capillary leakage and haemorrhage [13].

Management of EVALI, whether presenting as ALI or suspected DAH, relies on cessation of vaping and corticosteroid therapy tailored to the severity and suspected pathology [14,15]. For ALI-predominant EVALI, moderate-dose corticosteroids (e.g., prednisone 0.5-1 mg/kg/day) are typically employed to mitigate inflammation, often with supplemental oxygen or ventilatory support as required [14]. In contrast, suspected DAH may necessitate high-dose pulse corticosteroids (e.g., intravenous methylprednisolone 1,000 mg/day for three days), as administered in this case, to control alveolar bleeding, followed by an oral taper [15]. Prognostically, EVALI with ALI features often resolves fully with early intervention, though some patients experience persistent lung function deficits or interstitial lung disease [14]. DAH in EVALI, while less studied, appears to carry a favourable prognosis with prompt treatment and vaping cessation, as evidenced by this patient’s stability at three-month follow-up; however, untreated DAH risks severe hypoxemia or fibrosis, necessitating ongoing surveillance [15].

As there is no specific treatment for EVALI, the CDC recommends cessation of e-cigarette use and corticosteroid treatment, although no standard dose is advised [16]. Prognosis following steroid treatment varies significantly, ranging from complete resolution of clinical symptoms and radiographic abnormalities to the persistence of pulmonary disease, such as interstitial lung disease [4,16].

## Conclusions

The global rise in e-cigarette use, particularly among young individuals, has brought increased attention to EVALI, a heterogeneous condition that includes ALI and rare manifestations such as suspected DAH. This case highlights the importance of considering EVALI in patients with a history of vaping who present with haemoptysis and bilateral infiltrates, especially when other causes have been ruled out.

A detailed history, including the use of plant-based vaping products, is essential due to the non-specific nature of clinical and BAL findings. Early recognition, cessation of vaping and corticosteroid therapy can lead to favourable outcomes, though further research is needed to fully understand the spectrum of EVALI and guide optimal treatment strategies.
